# Cardioneuroablation: A Comprehensive Review

**DOI:** 10.31083/RCM48106

**Published:** 2026-05-26

**Authors:** Sergio Conti, Andrea Giuseppe Porto, Paolo Zappulla, Giuseppe Sgarito

**Affiliations:** ^1^Division of Cardiology, Department of Internal Medicine, Section of Cardiac Electrophysiology, The Carver College of Medicine, University of Iowa Health Care Center, University of Iowa, Iowa City, IA 52242, USA; ^2^U.O.C. di Cardiologia con UTIC ed Emodinamica, Ospedale Cannizzaro, 95126 Catania, Italy; ^3^Divisione di Cardiologia, Azienda Ospedaliero Universitaria Policlinico “G.Rodolico - San Marco”, 95123 Catania, Italy; ^4^IRCCS ISMETT-UPMC Heart Center, Institute for Transplantation and Advanced Specialized Therapies, 90127 Palermo, Italy

**Keywords:** syncope, sinus arrest, atrioventricular block, vasovagal syncope, cardioneuroablation

## Abstract

Cardioneuroablation (CNA) has emerged as a promising therapeutic strategy for functional bradyarrhythmias, particularly in cases of cardioinhibitory neurocardiogenic syncope and certain forms of atrial fibrillation. Indeed, by targeting vagal innervation through endocardial radiofrequency catheter ablation, CNA can obviate the need for pacemaker (PM) implantation. This technique involves denervation of specific vagal nerve structures within the atria to modulate autonomic balance and prevent symptomatic bradycardia. The efficacy of this approach stems from the recognition that an imbalance between sympathetic and parasympathetic tones, often characterized by excessive vagal activity, underpins these arrhythmogenic conditions. Indeed, CNA may be more effective than a permanent PM implantation in some patients, as this method addresses the underlying etiology rather than merely treating symptoms. Specifically, modulating autonomic nervous system (ANS) signaling through procedures such as CNA holds considerable promise for preventing and treating a range of cardiac arrhythmias. This review aims to synthesize current knowledge regarding various CNA techniques, exploring the associated mechanisms, clinical applications, and outcomes across diverse patient populations.

## 1. Background

Vasovagal syncope (VVS) is one of the most common forms of syncope, with a cumulative lifetime incidence estimated at 32% in men and 42% in women by age 60 [1]. It is triggered by an imbalance in the autonomic nervous system (ANS), characterized by increased vagal activity, which leads to bradycardia and/or peripheral vasodilation, ultimately causing transient cerebral hypoperfusion [2]. The clinical course of VVS is typically benign; however, up to 35% of patients experience recurrent episodes within a year, significantly impairing their quality of life and increasing the risk of injuries [3]. Standard management includes lifestyle adjustments, physical counterpressure maneuvers, and medications such as fludrocortisone or midodrine, which can carry several side effects, especially in young patients. Despite guideline-directed therapy (GDMT), about 20% of patients remain symptomatic [4]. Selected patients over 40 years of age who experience a prevalent cardioinhibitory form of VVS may benefit from dual-chamber pacemaker (PM) implantation [5, 6]. However, potential drawbacks associated with long-term device-related issues must be carefully considered.

Over the last two decades, cardioneuroablation (CNA) has emerged as a therapeutic option for functional bradyarrhythmias and cardioinhibitory VVS. This transcatheter ablation procedure targets the cardiac ANS by ablating to some extent the ganglionated plexi (GPs), which are clusters of intrinsic autonomic nerves predominantly situated in the epicardial fat pads of the atria. By doing so, CNA can suppress the vagal hyperactivity and restore autonomic balance [7, 8, 9]. Early clinical evidence suggests that CNA can provide promising results in carefully selected VVS patients with a prevalent cardioinhibitory response [7, 8, 10]. Nevertheless, several challenges remain unresolved, including the definition of optimal patient selection criteria and the establishment of standardized ablation protocols, which currently limit its broader application [11].

In this paper, we will review the existing CNA techniques for the treatment of cardioinhibitory VVS and functional bradyarrhythmias, starting from the cardiac ANS anatomical background. We will then report the outcomes of the most relevant studies, developments in the procedure, existing uncertainties, potential complications, and the future horizon of CNA.

## 2. Clinical Evidence

The vast majority of studies published so far are observational, non-randomized, and open-label. The only available randomized controlled trial published in 2023 was not blinded and included a small population of 48 patients with highly symptomatic VVS (24 treated with CNA vs 24 treated with optimal non-pharmacological therapy). Syncope recurrence rate at 2 years was 8% in the CNA arm compared with 54% in the control arm (*p* = 0.0004), with an excellent safety profile with no procedural complications reported [12]. A recent single-arm meta-analysis published in 2025 included 28 studies (observational in the vast majority but including also the aforementioned randomized trial) with a total of 1153 patients (median age 39.6 years, 51.86% female sex) with VVS, where CNA’s potential efficacy was tested [13]. The baseline median number of syncope occurrences over the previous year was 3.8. The syncope recurrence rate after the CNA was 5.94% (95% CI 3.37–9.01) with a median follow-up of 21.4 months. Syncope recurrence at 12 months was 2.61% (95% CI 0.45–5.87). The syncope recurrence rate was higher among patients treated with right atrial (RA) GP ablation only than with biatrial ablation (15.8% vs 4.4%). Recurrences were also more frequent in those patients in whom the procedure used only an electroanatomical map (EAM) to target the GP, compared with the combination technique of EAM plus fractionated electrogram (EGMs) or EAM plus high-frequency stimulation (HFS), 8.33%, 5.14%, and 4.04%, respectively. Finally, the prevalence of CNA procedure complications was 0.99% (95% CI 0.14–2.33), with most of them represented by groin hematomas and rarely by pericardial issues. Similarly, in the 2024 European Heart Rhythm Association (EHRA) statement on CNA in VVS, 28 papers were analyzed, and CNA efficacy ranged from 73.2% to 100% [14].

In summary, CNA showed a potential value, especially for patients with VVS. However, the design and quality of available contemporary studies still prevent us from determining the definite net clinical benefit of CNA compared with current GDMT (pacemakers included). Specifically, CNA efficacy might be overestimated due to a placebo effect, elevated spontaneous remission rates of VVS, and effective pharmacological and non-pharmacological therapies in addition to the CNA procedures performed in the studies. Finally, potential CNA side effects, such as long-lasting inappropriate sinus tachycardia (described mainly in case reports) or a post-CNA proarrhythmic state, were not specifically addressed by the majority of the studies considered so far [10]. A sham trial is currently ongoing, evaluating RA CNA in patients with neuromediated syncope (NCT04755101).

## 3. Anatomical Considerations

**Fig. 1.**
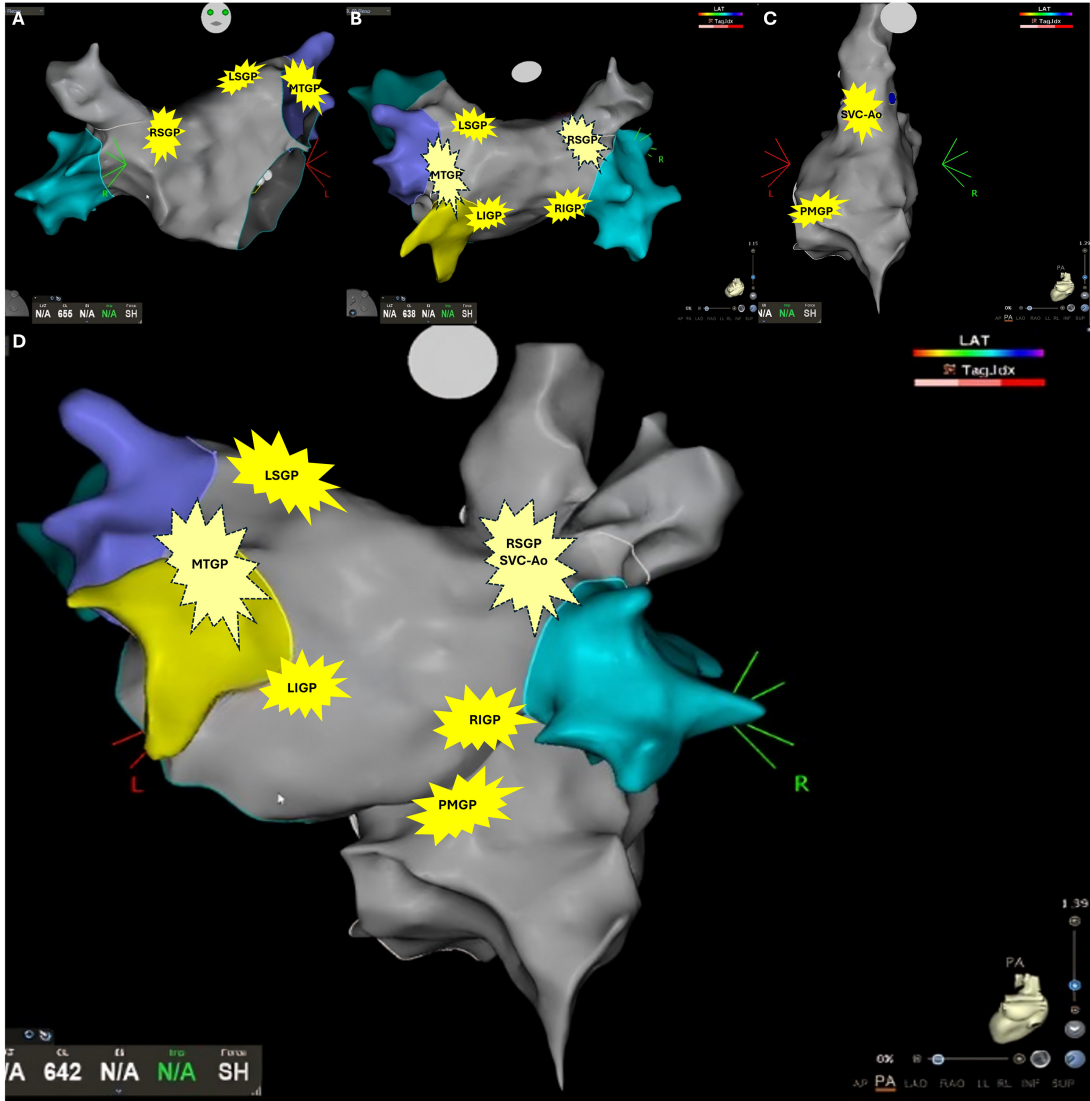
**Schematic localization of ganglionated plexi (GPs)**. (A) Right anterior oblique visualization of the left atrium. (B) Posterior visualization of the left atrium. (C) Posterior visualization of the right atrium. (D) Combined posterior visualization of the left and right atrium. Legend: RSGP, right superior GP; LSGP, left superior GP; MTGP, Marshall tract GP; LIGP, left inferior GP; RIGP, right inferior GP; PMGP, postero-medial GP; SVC-Ao, superior vena cava – aortic GP.

The ANS is classically delineated into extrinsic (spatially distant from the heart) and intrinsic (adjacent to the heart) components. Each of these systems plays distinct yet interdependent roles in regulating cardiac function. The extrinsic sympathetic system originates from preganglionic neurons in the spinal cord (T1–T5), which synapse in cervical and thoracic ganglia, most notably the stellate and middle cervical ganglia [15]. The resulting postganglionic fibers form extensive innervation through the cardiac plexus, penetrating deeply into the ventricular and atrial myocardium and endocardium, as well as the sinoatrial (SA) and atrioventricular (AV) nodes. Norepinephrine acts via β_1_-adrenergic receptors to enhance heart rate, conduction velocity, contractile strength, and relaxation. Complementarily, the parasympathetic system arises from the dorsal motor nucleus of the vagus and the nucleus ambiguus. Its fibers reach the heart via the vagus nerve (cranial nerve X), synapsing in intramural intrinsic GPs located within the epicardial fat pads. Short postganglionic fibers from these GPs primarily innervate the SA node, AV node, and atrial myocardium and endocardium. Activation of this pathway via acetylcholine on M_2_ receptors results in decreased heart rate, slowed AV conduction, and reduced atrial contractility. The postganglionic sympathetic fibers either directly innervate the myocardium or first synapse on the intrinsic cardiac GPs, while all preganglionic parasympathetic fibers synapse on the intrinsic cardiac GPs [16]. Atrial chambers are more densely innervated by cholinergic nerves, while the ventricles are mainly innervated by adrenergic fibers [17, 18]. The intrinsic cardiac nervous system (ICNS), which constitutes the network of the epicardial GPs, is mainly embedded in the atrial epicardium and follows a pattern of six to ten subplexuses [19, 20]. Most GPs are closely associated with the pulmonary veins (PVs) or with other vascular structures entrenched in the atrial wall, such as the coronary sinus (CS) or the superior vena cava (SVC). The ICNS serves as an autonomic integration hub for both sympathetic and parasympathetic modulation. GPs, in fact, are mainly constituted by interconnecting sympathetic and parasympathetic neurons and are a reservoir of a multiplicity of neuropeptides and neuromodulators, including calcitonin gene-related peptide, vasoactive intestinal polypeptide, and nitric oxide [21]. Landmark anatomical studies have delineated the main GPs (Fig. 1) [19]:

- the right superior GP (RSGP), near the SVC–right superior PV (RSPV) junction and in close proximity to the interatrial septum. From this ganglion, most of the efferent parasympathetic fibers travel into the atria through the medial part of the SVC and the aortic root. The RSGP location is usually quite consistent among individuals, and this GP predominantly influences SA nodal function [22];

- the right inferior GP (RIGP), which modulates AV nodal conduction;

- the posteromedial left atrial GP (PMLGP), which is located on the posteromedial surface of the LA around the ostium of the CS and, like the RIGP, modulates the AV node function;

- the left superior (LSGP) and left inferior GP (LIGP), which exert mixed nodal effects;

- the Marshall tract GP (MTGP), which is associated with the ligament of Marshall containing cholinergic fibers.

All these GPs form an interconnected network that enables autonomic cross-communication and regulation [23]. The larger GP can contain up to 400 neurons [20]. The peculiar location of the ICNS on the epicardial side of the thin-walled atria, which typically measure 3–4 mm in thickness, makes it accessible by radiofrequency (RF) ablation. In particular, the RSGP, RIGP, and PMLGP can be reached by RF application from the RA without the need to access the LA. These anatomical and functional insights provide the foundation for CNA. By targeting and ablating these epicardial GPs via an endocardial approach, the procedure reduces excessive vagal outflow, thereby correcting the autonomic imbalance that is central to cardioinhibitory VVS and related bradyarrhythmias [14]. First developed in 2005 by Pachon and colleagues [7], CNA is a technique designed to selectively disrupt parasympathetic pathways while preserving, or even enhancing, sympathetic dominance, thus restoring autonomic equilibrium. Since parasympathetic fibers regenerate slowly, CNA can provide sustained relief in the long term. In contrast, sympathetic regrowth occurs more rapidly, resulting in a selective long-term suppression of parasympathetic activity.

## 4. Cardioneuroablation for Cardioinhibitory VVS and Functional Bradyarrhythmias

Cardioinhibitory VVS and functional bradyarrhythmias are closely linked, both involving an overactive parasympathetic system causing bradycardia leading to syncope. Clinical characteristics are summarized in Table 1. The exact pathophysiologic mechanism of VVS is still unknown. Two main theories are described: (1) The “peripheral theory” and (2) The “central theory” [24]. In the “peripheral theory”, patients prone to excessive pooling of peripheral venous blood have a subsequent decrease in cardiac preload, stroke volume, and cardiac output. A reduction in blood pressure will activate the sensitive stretch baroreceptors in the blood vessel wall. Receptors that are under-stretched usually increase the sympathetic tone and diminish the parasympathetic activity. However, in VVS, the sympathetic tone is significantly reduced or abolished; therefore, heart rate and peripheral vasculature tone further decrease the cardiac preload. In volume-depleted ventricles, the myocardial contraction activates the ventricular mechanoreceptors (Bezold-Jarisch reflex), which are unmyelinated vagal afferent C-fibers. The final feedback response will send efferent parasympathetic signals to activate the postganglionic fibers, located in the para-cardiac GPs. In cardioinhibitory VVS, increased parasympathetic activity, combined with abolished sympathetic activity, reduces total peripheral resistance and cardiac automatism, leading to syncope from bradycardia, AV blocks, or sinus arrest. In the “central theory”, the reduction in the cerebral blood flow triggers the VVS symptoms. The cerebral blood vessels are innervated by regulatory mechanisms involving sympathetic and parasympathetic fibers, which decrease cerebral arteriolar vascular resistance to maintain brain perfusion when systemic blood pressure is reduced. In patients with VVS, a “paradoxical cerebral vasoconstriction” results from an increase in cerebrovascular resistance when systemic blood pressure decreases, leading to cerebral blood flow below the lower limit and syncope.

**Table 1.** **Clinical characteristics of vasovagal syncope and functional bradycardias**.

	Triggers	Prodromal symptoms	Sinus bradycardia	Atrioventricular block	Response to atropine
Vasovagal syncope	Present.	Present.	Present during the episodes.	Present during the episodes.	Effectively treats cardioinhibitory VVS. Less effective in vasodepressor.
	Prolonged standing, intense emotions/pain, medical procedures, excess heat, dehydration, bowel movements/urination, coughing.	Fatigue, warm feeling, profuse sweating, pallor, nausea, yawning, vision changes, and dizziness.	Precedes sinus pauses/arrest or asystole.	II-degree AVB, 2:1, or higher-degree AVB.	
Hypervagotonic sinus node dysfunction	Usually not present.	Usually not present.	SNRT, SACT, and chronotropic response are typically normal. Evidence of paroxysmal sinus pauses, sinoatrial block, and sinus arrest.	Occasionally associated.	Increase in sinus rate >25%. Normalization of SNRT and SACT, if prolonged.
	May be exacerbated by intense pain/emotions, intense physical activities, bowel movements/urination.	Symptoms reported during the bradycardia episodes: fatigue, weakness, shortness of breath, reduction in exercise capabilities, lightheadedness, dizziness, nausea, and syncope.			
Functional atrio-ventricular block	Usually not present.	Usually not present.	Occasionally associated.	Normal HV.	Resolution of AVB.
	May be exacerbated by intense pain/emotions, intense physical activities, bowel movements/urination.	Symptoms reported during the bradycardia episodes: fatigue, weakness, shortness of breath, reduction in exercise capabilities, lightheadedness, dizziness, nausea, and syncope.		Transition from normal conduction to advanced AVB is usually preceded by bradycardia.	

AVB, atrioventricular block; VVS, vasovagal syncope; SNRT, sinus node recovery time; SACT, sinoatrial conduction time; HV, His-ventricular.

**Fig. 2.**
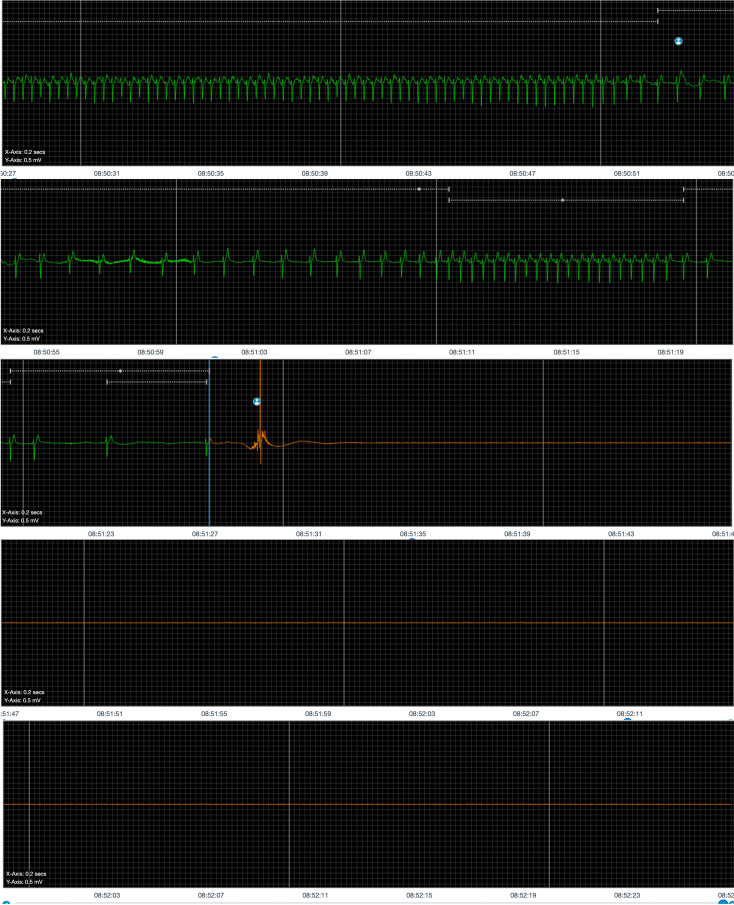
**Monitoring of a patient with severe hypervagotonic sinus node dysfunction (SND)**.

Functional bradyarrhythmias are a specific type of conduction disorder characterized by excessive parasympathetic activity, rather than by intrinsic or degenerative diseases affecting the SA or AV node. This excessive activity can lead to significant pauses in heart rhythm, sinus arrest, or atrioventricular block (AVB) (Fig. 2). The two primary forms associated with this mechanism are functional AVB and hypervagotonic sinus node dysfunction (SND). Traditionally, the standard treatment for severe bradyarrhythmias has been the implantation of a permanent PM. However, an increasing amount of evidence suggests that for carefully selected patients with a clear vagally mediated phenotype, CNA can effectively suppress harmful vagal reflexes, eliminate symptoms, and reduce the need for a PM in most cases. This approach has demonstrated a favorable safety profile and provides lasting symptom relief [14, 25, 26, 27, 28, 29]. This chapter will focus on the diagnosis and identification of suitable patients, which are essential for proper referral for CNA, and will critically evaluate the clinical outcomes associated with this treatment. Indications, diagnostic criteria, and expected outcomes are summarized in Table 2 (Ref. [14, 30, 31, 32, 33]).

**Table 2.** **Indications, diagnostic criteria, and expected outcomes of CNA**.

	Indications	Diagnostic criteria	Expected outcomes
Vasovagal syncope	Young patients (<40 years old) with severe forms of cardio-inhibitory VVS with frequent and debilitating symptoms, with limited prodromes, repeated injuries, and impaired quality of life. May be considered as an alternative to pacing in patients aged >40 years old in selected cases.	At least one documented episode of spontaneous cardio-inhibitory VVS, ECG/Holter/ILR evidence of spontaneous asystolic syncope, or symptomatic >3 s asystolic pause or significant bradycardia with HR <40 bpm provoked during a tilt-table test,	The primary outcome is freedom from syncope or significant reduction in syncope recurrence. Secondary outcomes are currently based on periodical resting ECG, Holter ECG/ILR, and HRV evaluation. EHRA/HRS/APHRS/LAHRS Scientific Statement [14].
		AND	
		a positive response to atropine challenge (>25% increase in sinus rate after 0.04 mg/kg for patients <50 kg or 2 mg if >50 kg).	
Hypervagotonic sinus node dysfunction	Young and highly symptomatic intermittent or persistent sinus bradycardia documented on prolonged ECG monitoring/ILR with frequent, debilitating symptoms and impaired quality of life.	Intermittent or persistent sinus bradycardia on prolonged ECG monitoring/ILR, without typical. clinical history for cardio-inhibitory VVS, AND symptomatic cardio-inhibitory pauses ≥3 s or asymptomatic ≥6 s, or progressive sinus bradycardia preceding hypotension during tilt-table test,	Substantial increase in resting sinus rate, freedom from syncope, and avoidance of pacing. U.S. multicenter registry reported outcomes in the SND subgroup comparable to the overall cohort [33].
		AND	
		a >25% increase in sinus rate after atropine challenge, normalization of SNRT and SACT, if prolonged.	
Functional atrio-ventricular block	Young and highly symptomatic intermittent or persistent AVB documented on prolonged ECG monitoring/ILR with frequent, debilitating symptoms and impaired quality of life.	At least one syncopal episode and documentation of daytime second or third-degree AVB,	Atrio-ventricular conduction normalization, freedom from syncope, avoidance of pacing [30, 31, 32].
		AND	
		normalization of atrio-ventricular conduction during atropine challenge.	

CNA, Cardioneuroablation; ILR, implantable loop recorder; HR, heart rate; HRV, heart rate variability; EHRA, European Heart Rhythm Association; HRS, Heart Rhythm Society; APHRS, Asia Pacific Heart Rhythm Society; LAHRS, Latin American Heart Rhythm Society; SND, sinus node dysfunction.

### 4.1 Functional Atrioventricular Block

#### 4.1.1 Patient Identification and Diagnosis

**Fig. 3.**
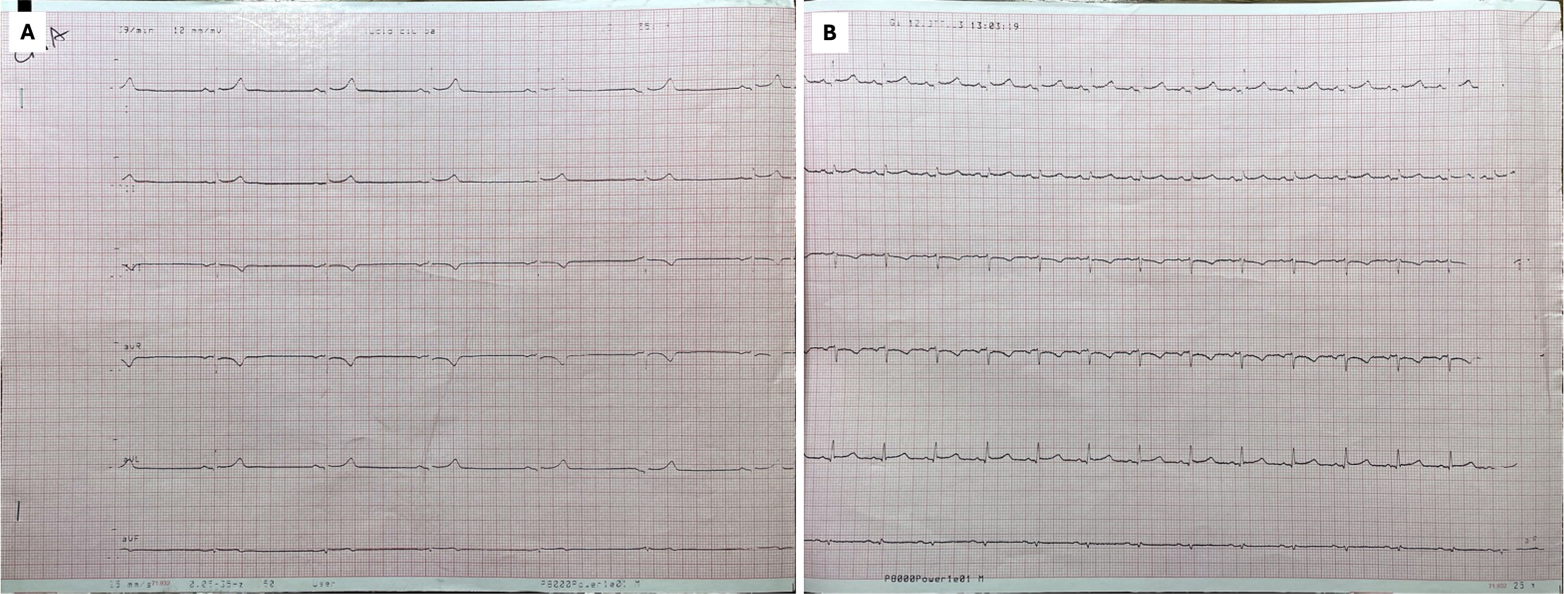
**12-lead ECG before (A) and after (B) atropine challenge**. A significant increase in heart rate is evident.

Functional AVB typically occurs in young or middle-aged patients with structurally normal hearts. These patients experience sudden, paroxysmal episodes of high-grade or complete AVB that are fully reversible. Episodes often occur at night, after meals, or are triggered by reflex syncope. They are frequently accompanied by sinus slowing, indicating a strong vagal influence. Holter or implantable loop recorder (ILR) monitoring typically reveals the sudden transition from normal conduction to advanced AVB, followed by spontaneous recovery. The atropine challenge is a reliable screening test for patients who may benefit from CNA. A heart rate increase of more than 25% or exceeding 90 bpm, and/or resolution of complete AVB after atropine administration, strongly suggests a vagally mediated mechanism (Fig. 3). Electrophysiological studies (EPS) can help differentiate functional from intrinsic disease; a normal HV interval (<70 ms) supports a functional cause. Head-up tilt testing (HUT) adds to the evaluation; positive findings may include asystole of 3 or more seconds with symptoms, a sinus/AV pause of 6 seconds or more without symptoms, or vagal AVB with sinus slowing or bradycardia preceding hypotension. Pure vasodepressor responses without significant bradycardia typically do not warrant a CNA procedure.

#### 4.1.2 Clinical Evidence and Outcomes

**Fig. 4.**
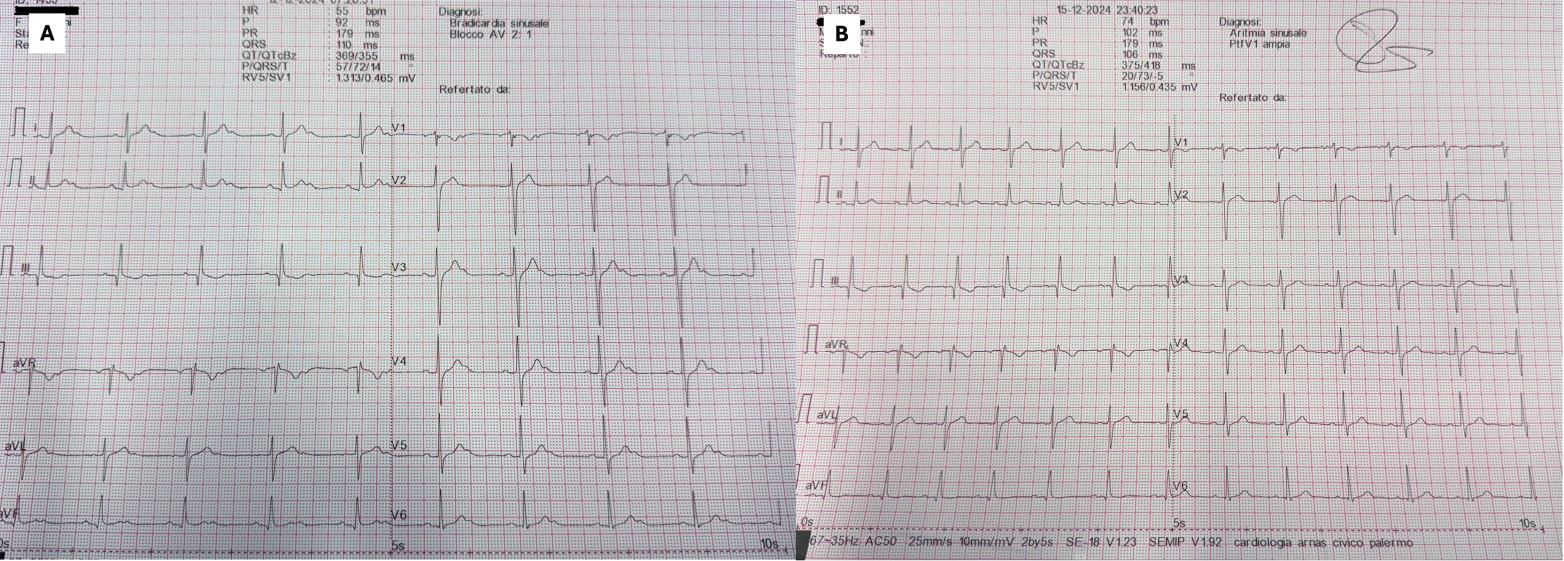
**12-lead ECG of functional atrio-ventricular block before (A) and after (B) CNA**.

The early Brazilian experiences demonstrated that CNA can eliminate vagally mediated AVB and prevent syncope, achieving an acute procedural success rate of over 90%, enabling long-term PM avoidance in the majority of patients [7, 25, 26] (Fig. 4). These results have been confirmed in larger and more diverse cohorts. Aksu *et al*. [30] first described CNA as a therapeutic approach to functional AVB. In this population, they reported a substantial reduction in recurrence of functional AVB episodes after CNA during follow-up with avoidance of pacemaker [30]. Subsequently, the PIRECNA multicenter registry, which included 130 patients, reported an acute success rate of 96% and only a 14% recurrence rate of bradyarrhythmia at a median follow-up of ten months, with more than 85% of patients avoiding pacemaker implantation despite initial indications for it [31]. A single-center prospective cohort study conducted in China, involving 60 participants, reported a 95% acute success rate, approximately 10% recurrence at one year, and no major complications [34]. Additionally, mechanistic case series have shown that CNA can eliminate late functional AVB occurring after atrioventricular nodal reentrant tachycardia (AVNRT) ablation, with no reported recurrences during follow-up [32]. The largest contemporary dataset, a U.S. multicenter registry including 205 patients, documented an average increase of 20 bpm in resting sinus rate, 78% freedom from syncope, 97% avoidance of pacemaker implantation, and a major complication rate of only 1.4% at a median follow-up of 14 months [33].

The strength of these results is further supported by data on VVS, a disorder characterized by the same vagal mechanism. In VVS, a randomized controlled trial involving 48 highly symptomatic patients showed a two-year syncope recurrence rate of only 8% in the CNA group compared to 54% in the control group (*p* = 0.0004), and there were no procedural complications [12]. A recent meta-analysis of 28 studies with 1153 patients reported a pooled syncope recurrence rate of 5.94% (95% CI 3.37–9.01) at a median follow-up of 21 months, with a 12-month recurrence rate of 2.61% and a procedural complication rate of less than 1%, mostly consisting of minor vascular events. Outcomes were significantly better with biatrial ablation compared to right-sided-only procedures (recurrence: 4.4% vs 15.8%) and when combined anatomical and functional targeting was used, yielding recurrences of 4–5% compared to anatomical mapping alone (8.3%) [13]. The EHRA 2024 consensus statement corroborates these findings, citing the CNA efficacy of 73.2%–100% across published VVS series [14]. Although most comparative data derive from VVS, the shared vagal mechanism provides compelling indirect evidence that CNA can safely and effectively treat functional AVB, with high rates of acute success, durable symptom control, and very low complication rates, making it a viable alternative to permanent pacing in appropriately selected patients.

### 4.2 Hypervagotonic Sinus Node Dysfunction

#### 4.2.1 Patient Identification and Diagnosis

Hypervagotonic SND is a significant clinical concern in patients, particularly young individuals who are otherwise healthy. This clinical entity overlaps with patients with cardioinhibitory VVS. The diagnosis is based on clinical history and can be confirmed by evaluating the sinus rate response to atropine infusion. In the case of hypervagotonic SND, atropine infusion results in resolution of the bradycardia. These patients often experience recurrent fainting spells or prolonged pauses in their heart rate, which can occur during sleep or in reflex situations, despite having normal sinus function at baseline and maintaining a healthy chronotropic response outside of vagal episodes. Prolonged ECG monitoring or ILR documentation of paroxysmal sinus arrest or severe bradycardia provides diagnostic confidence. A positive atropine test and normal exercise chronotropic response further support a vagal mechanism. EPS typically shows normal sinus node recovery time (SNRT) and sinoatrial conduction time (SACT). Similar to AVB, HUT can reveal cardioinhibitory pauses ≥3 seconds with symptoms or ≥6 seconds without symptoms, or progressive sinus bradycardia preceding hypotension. These patterns help differentiate hypervagotonic SND from intrinsic sinus node disease [14].

#### 4.2.2 Clinical Evidence and Outcomes

Evidence supporting CNA in hypervagotonic SND has grown from early mixed cohorts, prospective series, multicenter registries, and mechanistic case reports. Although the literature is smaller than that for functional AVB, the direction and magnitude of benefit are remarkably consistent when a vagally mediated mechanism is rigorously documented. Initial mixed experiences indicated that targeting atrial GPs can restore sinus node function, resulting in high acute success rates and lasting symptom relief during follow-up, with most patients avoiding PM implantation [7, 25, 28]. Prospective single-center studies have confirmed these findings. When a hypervagotonic phenotype was rigorously validated—using ILR documentation of paroxysmal sinus arrest, a positive atropine test, preserved chronotropic reserve, and normal SNRT/HV intervals—the rate of acute success approached 95–100%. This was accompanied by significant reductions in pause burden and sustained freedom from syncope during mid-term follow-up [34]. The U.S. multicenter registry reported outcomes in the SND subgroup that were comparable to the overall cohort. The results showed a substantial increase in resting sinus rate (approximately +20 bpm), about 80% freedom from syncope, approximately 95–97% avoidance of PM use, and a major complication rate of about 1–2% at around 14 months [33].

In patients with structurally normal hearts and positive atropine responses, targeted denervation—often starting at the RSGP near the SVC/RSPV—resulted in immediate and sustained sinus acceleration, elimination of pauses, and maintained freedom from symptoms for 6–12 months [35]. Anatomical and neuromodulation studies support these observations, emphasizing the dense vagal innervation of the sinus node region and reinforcing the idea that strategic denervation at “gateway” plexi can restore autonomic balance in the sinus node.

Pediatric data, though necessarily limited, are particularly noteworthy. In pediatric patients with vagally mediated SND (some also exhibiting functional AVB), CNA achieved complete symptom resolution and PM avoidance after one year. Given the long-term risks associated with pacing in young patients (such as lead failure, infection, and the need for multiple generator replacements), these results are clinically significant and underscore the value of CNA when a hypervagotonic phenotype is clearly documented [36, 37, 38].

In hypervagotonic SND, the recurrence rate of bradyarrhythmia is generally low, around 10–20% over 1 to 2 years, and is often linked to autonomic reinnervation. While re-ablation may occasionally be necessary, most relapses are partial and can be managed without the need for PM implantation. Major complications are rare, occurring in about 1–2% of cases, with the most common functional side effect being inappropriate sinus tachycardia (IST), which is typically self-limiting or easily managed with medication [39]. Although there is a lack of randomized evidence specific to SND, we can cautiously infer from studies on VVS due to shared vagal physiology. A randomized controlled trial in VVS showed a two-year recurrence rate of syncope of 8% with CNA compared to 54% with standard care, with no procedural complications reported. Additionally, a meta-analysis of 28 studies involving 1153 patients indicated a pooled recurrence rate of approximately 6% over roughly 21 months and a complication rate of less than 1%. The analysis found that biatrial ablation and combined anatomical and functional targeting outperformed single-atrium or purely anatomical strategies [13]. Overall, these findings support CNA as an alternative treatment option for hypervagotonic SND, particularly in younger patients, for whom permanent pacing could pose long-term device-related risks.

## 5. Safety, Clinical Implications, and Perspectives

We acknowledge that most data on CNA are from retrospective or prospective non-randomized studies. However, across all cohorts, CNA for cardioinhibitory VVS, functional AVB, and hypervagotonic SND demonstrates a favorable safety profile. Major complications are rare, with rates around 1–2% in large registries [33]. The most common functional adverse effect is IST, which arises from excessive vagal withdrawal; however, IST is typically self-limiting or can be effectively managed with medication. However, long-term burden, impact on quality of life, and its management are not fully explored. The potential risk of tachycardiomyopathy should be considered. However, CNA is not the only ablation procedure that may cause IST. Similarly, heart rate acceleration has been described in patients undergoing thermal AF ablation. It is not surprising because several areas where RF energy is delivered during PV isolation are the same as those ablated during CNA, especially at the anterior ridge of the RSPV. It has been shown that the extent of parasympathetic denervation after AF ablation is similar to that occurring after CNA, and several reports suggest the increase in sinus rate to be a good predictor of AF ablation efficacy. Nonetheless, no increase in the rate of complications associated with faster heart rate nor elevated risk of death following AF ablation has been reported so far, which is encouraging when the risk of IST after CNA is evaluated [40, 41].

Recurrence of bradyarrhythmia happens in about 10–20% of patients, often due to autonomic reinnervation. While some cases may necessitate repeat ablation, many can be treated conservatively. From a clinical standpoint, the most significant implication of CNA is its ability to prevent the need for permanent pacing in most appropriately selected patients. This is particularly beneficial for younger and pediatric populations, who might otherwise require decades of device therapy along with its associated risks [14]. These findings establish CNA as a safe and effective alternative to PM implantation when the vagal mechanism is clearly documented and intrinsic disease has been ruled out. Standardizing diagnostic criteria—such as tilt-test thresholds, atropine response, and ILR documentation—remains a priority. Additionally, randomized controlled trials are needed to confirm long-term efficacy and durability. Until such evidence becomes available, the current multicenter and meta-analytic data provide a strong foundation for recommending CNA to patients with well-documented vagally mediated bradyarrhythmias.

## 6. Different CNA Approaches and Techniques

### 6.1 Approach #1: CNA Controlled by Extra-Cardiac Vagal Stimulation

In their first report of CNA procedure, Pachon *et al*. [25] described an ablation procedure guided by standard electrophysiological parameters evaluation, such as reduction in SNRT, increase of sinus rate, reduction in Wenckebach’s cycle length (WCL), and the evaluation of the response to atropine administration at the end of the ablation. However, these measurements may often be unreliable, influenced by sympathetic and parasympathetic balance or by medication used during general anesthesia, muscle relaxation, or mild sedation. To overcome these limitations, Pachon *et al*. [42] introduced a technique called extracardiac vagal stimulation (ECVS). This method offers a more accurate measurement of the vagal effect before, during, and after CNA [2]. ECVS provides a systematic, stepwise, and objective assessment of the acute efficacy of CNA. To perform ECVS, a decapolar steerable electrode catheter is advanced under fluoroscopic guidance through the SVC and the internal jugular vein near the right or left jugular foramen, which is the location nearest to the vagus nerve. Stimulation is performed using a neurostimulator that delivers a pulsed electric field within the jugular vein. At baseline, ECVS causes sinus arrest and atrioventricular node (AVN) conduction block. After CNA, these effects disappear, confirming successful vagal denervation induced by the procedure. ECVS is currently the only reliable tool available to assess the acute efficacy of CNA. Recently, a prospective study with up to 5 years of follow-up demonstrated that ECVS-controlled CNA resulted in a 4-fold reduction in symptom recurrence compared with empirical CNA [43]. This approach provides a more objective, rational, and systematic assessment, allowing targeted treatment of three distinct vagal innervation territories based on clinical indications: the sinus node, AVN, and atrial walls. These territories or *domains* are somewhat independent, exhibiting different patterns of innervation and denervation. Characterization of these domains is performed with ECVS. Sinus node innervation (*domain 1*) is simply proved by applying ECVS, causing sinus arrest. AVN innervation (*domain 2*) is demonstrated by the induction of high-degree AVB caused by ECVS. Atrial wall innervation (*domain 3*) is also tested with ECVS, utilizing the Vagal AF Induction Test (VAFIT) protocol and measuring Effective Atrial Refractory Period (EARP) shortening. Depending on the specific clinical indication, CNA may target one or more domains, each with a unique topography. Typically, for cardioinhibitory VVS and carotid sinus syndrome, denervation of *domains 1* and *2* is generally indicated. In hypervagotonic SND in the absence of AVB, denervation of *domain 1* is selectively targeted. In cases of functional AVB, AVN denervation is prioritized (*domain 2*), which is evaluated with ECVS applied to the left vagus nerve. Using this technique, CNA proceeds incrementally to eradicate vagal effects in the designated region (*domain*), terminating upon confirmed efficacy to preclude over-ablation [44].

The impact of ECVS on the efficacy of CNA remains uncertain. Comparative data between ECVS and conventional electrophysiological (EP) parameters as endpoints for CNA are limited. No randomized, prospective studies have addressed this question; available evidence is restricted to retrospective observational studies, which may only generate hypotheses. Recent studies reported a trend toward improved outcomes with ECVS. When data are combined, ECVS appears to be associated with a higher rate of freedom from syncope recurrence (91% versus 82%) [29, 43, 45]. Published evidence suggests that ECVS is particularly valuable for patients with reflex syncope due to functional AVB, as ablation in these cases is typically more extensive, requiring a bi-atrial approach that includes the inferior septal GPs, CS, and regions near the left PVs, in contrast to patients experiencing isolated sinus pauses [46, 47].

### 6.2 Approach #2: CNA Guided by Fractionated EGM Analysis

Although Armour *et al*. [19] described possible anatomical locations of major atrial GPs in human postmortem specimens, precise localization during CNA remains essential for targeted and circumscribed ablation. Several mapping techniques have been used, including endocardial HFS, visual assessment of fractionated EGMs, and anatomical approaches with 3D-electroanatomical mapping systems [48]. Each method, however, has notable limitations.

**Fig. 5.**
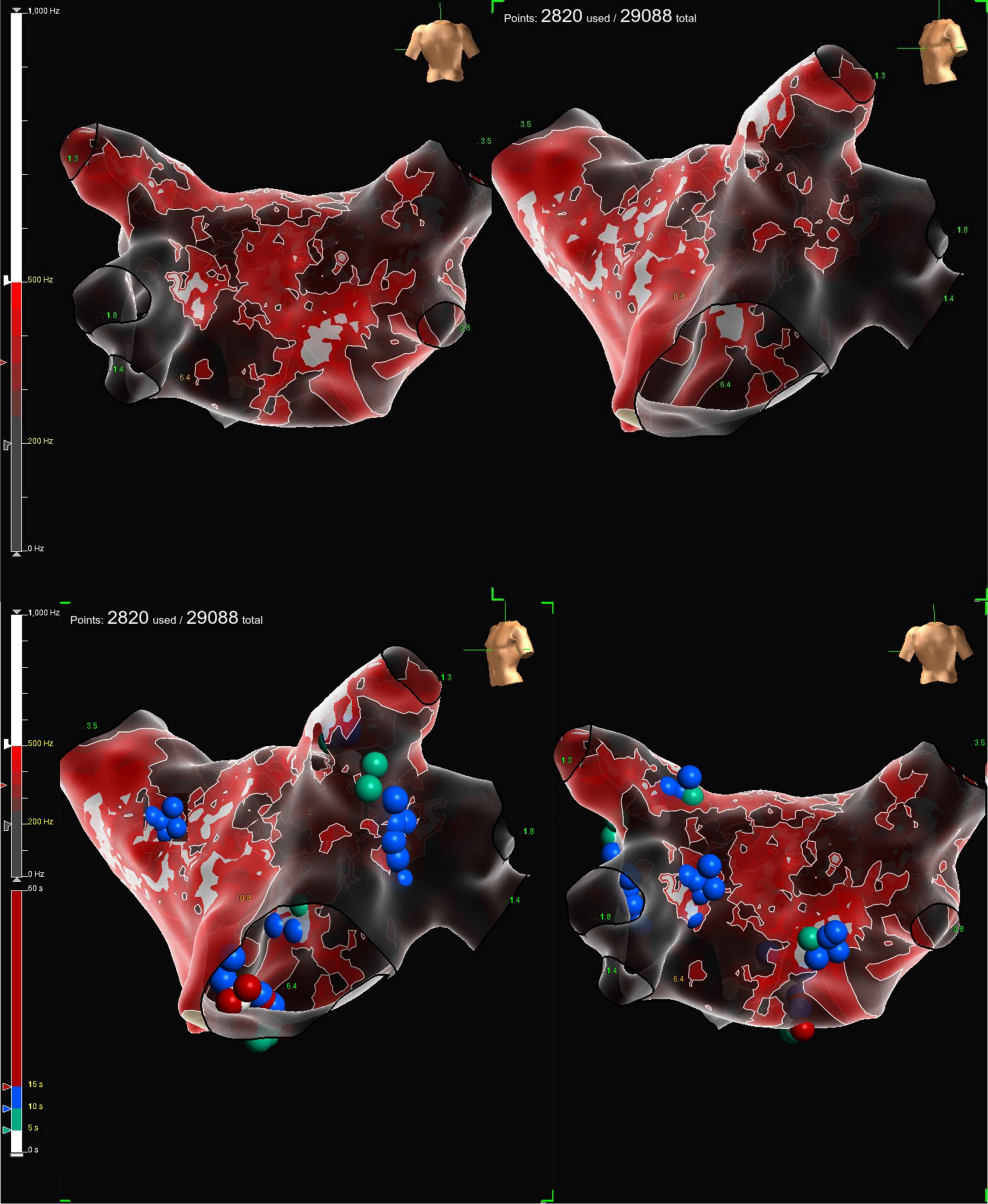
**Fractionation EGMs map of the left atrium during cardioneuroablation (CNA)**. The use of Omnipolar Technology Near-Field (OTNF™) with peak frequency of 550 Hz (white areas) is useful to identify target region for ablation. Blue, green, and red dots represent the ablation lesions on the Left Superior GP, Marshall Tract GP, Left Inferior GP, Right Superior GP, and Right Inferior GP. Image from Conti S, Sgarito G. Omnipolar Technology Near Field to Evaluate Anatomic Location of Ganglionated Plexi During Cardioneuroablation. *Clinical Case Reports*, 2026; 14: e72311 [52].

The GPs mapping technique guided by fractionated EGM was initially described by Pachon *et al*. [25], which identifies autonomic innervation areas by highly fractionated atrial EGMs, while the surrounding atrial tissue shows normal or less fractionated EGMs. Fast Fourier transform analysis categorizes the atrial myocardium is classified into two distinct types: (1) “*compact*” myocardium or normal atrial tissue, which displays dominant frequencies of ~40 Hz and a uniform spectral profile due to tightly interconnected cardiomyocytes, and (2) “*fibrillar*” myocardium or autonomic innervation areas, marked by fractionated EGMs, heterogeneous conduction, and frequencies >100 Hz arising from intramural neural and vascular elements. By changing high-pass filters on the EP recording system (200–500 Hz instead of the conventional band-pass filter settings of 30–500 Hz) during sinus rhythm, it is possible to target all EGMs that displayed multiple deflections (≥3) at the anatomical GP locations. This EGM-guided approach showed an equivalent efficacy in preventing prodromal symptoms and syncope recurrence compared to the prior method of combining HFS and spectral analysis [49]. Lellouche *et al*. [50] first analyzed EGM characteristics in relation to vagal responses during RF delivery and concluded that ≥4 EGM deflections can be used to identify autonomic innervation areas. Combining fast Fourier transform analysis and HFS for mapping and identification of GPs has also been reported [28]. However, human decisions based on visual interpretations of EGMs for fractionation may be poorly reproducible, particularly among less experienced operators. To enhance reproducibility, fractionation mapping software embedded in 3D-electroanatomical mapping systems can be used [26, 51]. In particular, the EnSiteX™ 3D-electroanatomical mapping system and the Advisor™ HD Grid SE™ multipolar mapping catheter can provide a fractionation mapping of both atria (Abbott, Minneapolis, MN, USA). This mapping is conducted using specific parameters: a width of 5 msec, a refractory time of 30 msec, a roving sensitivity of 0.1 mV, and a fractionation threshold >2. The color scale for the map was adjusted to designate areas at or above the fractionation threshold of >2 as white, indicating potential fractionated EGMs. These white areas were deemed as potential targets for ablation. In addition, a specialized combination of fractionation mapping software and peak frequency (PF) mapping software using the EnSite OT Near Field™ (OTNF™) algorithm can determine the ablation targets more precisely. This algorithm can differentiate between near-field and far-field components and identify the PF associated with the mapped intracardiac EGMs. The PF mapping using the EnSite OTNF™ algorithm can be applied on the same fractionation map, focusing on PF detection in the near field at 550 Hz (Fig. 5 (Ref. [52]) and Video. 1). The intersection of regions marked in white on the fractionation mapping with areas that had a PF greater than 550 Hz highlighted areas likely corresponding to the localization of GP, which were tagged as ablation targets. In cases where there was a poor correlation between the two maps in the indicated regions, areas showing a PF greater than 600 Hz were prioritized. Current approaches integrating fractionation EGM mapping software and PF can effectively delineate optimal ablation targets in each GP area and assist less experienced operators in focusing their mapping efforts to localize GPs accurately.

**Video 1.** **Vagal response during radiofrequency delivery in the area of the left superior ganglionated plexus (LSGP)**. Video associated with this article can be found, in the online version, at https://doi.org/10.31083/RCM48106.

Additionally, the use of a different software to identify GP location has been reported: the complex atrial fractionated electrograms (CFAE) software embedded in the CARTO3 3D-electroanatomical mapping system (Biosense, Webster, Inc., Diamond Bar, CA, USA). The target signal was characterized by >3 deflections, referred to as confidence interval levels (ICL) on the system, lasting at least 2 ms each, and an amplitude above 0.06 mV. Since the system acquires points by analyzing 2.5 seconds (2 seconds before and 0.5 seconds after the reference), the total number of ICLs is strongly dependent on the heart rate (HR) within this time frame. The settings for the color bar confidence level extremities should be established, considering the number of beats every 2.5 seconds, the desired minimum number of ICL, and the ventricular deflection in the areas surrounding the mitral and tricuspid valves. However, it must be considered that this feature, whose intention was to map AF, interferes with the accuracy of the window of interest, including the ventricular far fields in the counts. A duration value interval ranging from 2 msec to 140 msec has been chosen to include rapid deflections of the fragmented activity and to be as inclusive as possible. The selected threshold values ranged from 0.06 mV, to exclude noise and necrotic areas, to 1.5 mV, to avoid ventricular far-field signals. The color bar limits were set from 15 to 20, eliminating the less fragmented signals as areas with more than 7 deflections were targeted [53].

To date, there are no studies demonstrating that any software is superior to the visual analysis of EGMs. Large, multicenter randomized studies are needed to confirm any advantages of different approaches.

### 6.3 Approach #3: Anatomical Approach to CNA

In the past, anatomical studies have shown that most human atrial neurons are located between the origins of the two vena cavae. Armour *et al*. [19] identified ganglia on the posterior surface of the two atria and in the interatrial septum, noting a particularly high concentration of atrial epicardial GPs at the level of the Waterston’s groove. Since the 1990s, extensive studies have been conducted to identify the intrinsic components of the cardiac ANS, which include neurons situated on the epicardial surface, generally embedded within the epicardial fat pads. The anatomy and physiology of the ICNS are still being elucidated [20, 54, 55, 56]. However, it has been clearly demonstrated that cardiac autonomic ganglia are organized into discrete epicardial regions, termed GPs. Nerve fibers from these GPs directly innervate the SA and AV nodes, along with numerous other atrial and ventricular areas [7, 35, 48, 54, 55]. Notably, while GPs are primarily situated in the epicardium, extensive networks of afferent and efferent nerve fibers are also present at myocardial and endocardial levels [19, 20]. In aggregate, the human heart is estimated to harbour approximately 14,000 neurons within these ganglia [19]. Importantly, the autonomic innervation of the SA node and AVN is distinct. This separation enables tailored ablation strategies to address different types of cardio-inhibitory responses, such as sinus arrest and functional AVB [57]. In particular, the junction between the SVC and the septal aspect of the right superior pulmonary vein (RSPV) contains the greatest density of epicardial GPs innervating the SA node [20, 58]. Differently, the endocardial nerve fibers projecting from the epicardial GPs to the AVN are smaller and less distinctly defined. The primary GP area, known as the infero-septal GP, is typically situated in the pyramidal space between the CS ostium and the septal side of the inferior vena cava (IVC) and right inferior pulmonary vein (RIPV). Mechanistically, selectively ablating the septal GPs, including the RSGP and the inferior-septal GP, is generally enough to obtain the requisite denervation. This is consistent with clinical evidence indicating that targeted ablation of the inter-atrial septal GPs adjacent to the right PVs produces reliable vagal denervation of the SA and AV nodes [7, 59, 60]. The anatomical approach to CNA has been reported by Rivarola *et al*. [61, 62], reporting an efficacy of >70–80% in eliminating recurrent syncope and pre-syncope crisis. The ablation is performed under general anaesthesia without paralytics, enabling high-output pacing to localize the right phrenic nerve, which may course adjacent to target areas in the lateral RA. A detailed reconstruction of the RA’s anatomy—including both the SVC and IVC—and the LA, along with the PVs and CS anatomy, is necessary. This anatomical reconstruction is usually performed by using multipolar mapping catheter or mapping/ablation catheter. The septal GPs are identified based on their anatomical distribution. The RSGP is located in the region bounded by the septal aspect of the RSPV, the septal surface of the SVC at its junction with the RA, and the inferior margin of the right pulmonary artery. The inferior-septal GP is located within the area delineated by the proximal third of the CS and the septal aspect of the RIPV and IVC-RA junction. High-frequency fragmented multicomponent EGMs are characteristically recorded in these regions, signifying proximity to the GPs [7, 26, 35, 43, 48]. In addition to anatomical reconstruction, ECVS can be performed as described by Pachon and colleagues [42]. Recently, image integration has been increasingly used to accurately localize GPs. The use of cardiac computed tomography (CT) allows the identification of the fat pad in which GPs are located. Benabou *et al*. [63] reported a large interpatient variability in the anatomy of epicardial fat pads during CT-guided fat segmentation. In addition, the authors compared several target areas based on different ablation approaches (fractionated EGMs, anatomic, and CT-based). They found a limited correlation with EGMs fragmentation analysis and CT-based scan, resulting in inaccurate localization of GPs. The study concluded that an anatomical-based approach was more accurate to localize GPs and permitted a more targeted ablation strategy in patients for whom CT was not available [63].

In conclusion, ECVS, fractionated EGM analysis and mapping, and anatomical approach can be used as stand-alone techniques to guide CNA, or they can be integrated to increase the efficacy and safety of the procedure, since no method has demonstrated superiority in randomized comparative trials, and reproducibility and operator dependency remain key limitations.

## 7. Cardioneuroablation for Atrial Fibrillation

The role of the ANS as a trigger for the initiation and maintenance of atrial fibrillation (AF) is well established. Coumel *et al*. [64] firstly reported in a small case series of 18 patients without structural heart disease who had recurrent paroxysms of AF and atrial flutter, which appeared to be initiated by sinus rate slowing and atrial coupling attributed to vagal overactivity. Derangements in sympathetic tone are also thought to play a central role in AF, possibly via cellular, structural, and electrical changes that occur in the setting of states of heightened adrenergic tone.

Several clinical trials have explored the use of GP ablation in the management of AF. As a stand-alone treatment strategy for AF, GP ablation success rates have been poor. In one study examining the long-term impact of GP ablation during a 3-year follow-up period showed that isolated GP ablation was associated with significantly lower rates of freedom from atrial arrhythmias without antiarrhythmic drug therapy when compared to circumferential pulmonary vein isolation (PVI) (34.3% versus 65.7%, *p* = 0.008) [65]. As an additional strategy to PVI, the results of these studies have been favorable, with decreased rates of AF recurrence when compared to PVI alone [66, 67]. However, there is no standardized method for performing these ablations. Several pooled analyses, including an RCT-only meta-analysis, showed that GP ablation as an additional strategy to PVI may be more beneficial in patients with paroxysmal rather than persistent AF [68]. It has been hypothesized that nerve regeneration and reinnervation post-ablation may limit the durability of GP ablation on freedom from AF. However, given that additional GP ablation plus PVI has been demonstrated to be more effective than PVI alone, the nerve regeneration hypothesis may not be universally true, or it may suggest that additional factors independent of nervous inputs may be involved in this population and warrant further investigation.

## 8. Gaps in Evidence and Future Directions

There is increasing enthusiasm in the cardiac electrophysiology community regarding CNA. However, there are several grey zones that need to be further evaluated. First, we have data from studies with relatively small sample sizes and short follow-up durations. Second, various techniques are utilized for CNA, which include targeting specific areas (right atrial, left atrial, or biatrial), employing different ablation strategies (anatomical versus GPs identification), and defining procedural endpoints. Interprocedural variability is likely due to different procedural investigations aimed at determining which patients benefit most from each approach. However, as the evidence base strengthens, it will be crucial to establish relatively standardized approaches and reach a consensus on both immediate and clinically significant long-term endpoints. This is particularly important for the design and interpretation of randomized controlled trial results.

Additionally, CNA results in significant attenuation of cardiac parasympathetic tone that manifests as increased mean HR and decreased heart rate variability (HRV) [25, 26]. Reduced HRV and low baroreflex sensitivity have been shown to predict mortality following a myocardial infarction, independently of conventional risk factors [69]. In cases of myocardial infarction, congestive heart failure, and left ventricular dysfunction, reduced HRV also predicts both sudden and non-sudden cardiac death [70]. Additionally, maintaining intact cardiovagal innervation may help reduce infarct size and the incidence of ventricular arrhythmias after myocardial ischemia [71]. Recent studies in animal models have demonstrated that the ablation of cardiac cholinergic neurons increases susceptibility to ventricular arrhythmias. This heightened risk is likely due to the suppression of the cardioprotective effects of vagal innervation following central nervous system damage [72]. Given the possible detrimental long-term effects of CNA, the possibility of placebo effects should also be addressed. In patients with VVS, parasympathetic overdrive is a transient phenomenon. The risk of extensive and unnecessary denervation to treat transient vagosympathetic imbalance cannot be overlooked.

Finally, there has been some discussion regarding the need for a randomized, sham-controlled, double-blind clinical trial to evaluate the true efficacy of CNA in VVS [73]. According to previous studies, patients with recurrent syncope may be prone to medical or device placebos. Permanent pacing was associated with a reduced risk of recurrent syncope in unblinded studies and in studies comparing different pacemaker algorithms. However, no significant effect was observed in double-blinded trials. Awareness of having a functional, permanent pacemaker was associated with a substantial “*expectation*” effect, which independently reduced the risk of recurrent syncope [74]. Indeed, a sham trial is currently ongoing, and it will provide more insight into the real effects of CNA (NCT04755101).

In addition to syncope recurrence and freedom from PM as outcomes of ongoing and future registries and trials, it will be necessary to include quality of life (QoL) measurements and patient-reported outcomes to further evaluate the efficacy and impact of CNA. Some findings suggest that CNA is related to an improvement in QoL in patients with VVS [11]. The CNA for the Management of Patients with Recurrent Vasovagal Syncope and Symptomatic Bradyarrhythmias (CNA-FWRD) Registry is a multicenter prospective registry evaluating acute and long-term outcomes of VVS and AVB patients treated by conservative therapy and CNA [75]. In this registry, data regarding the impact of syncope on QoL will be collected before and after CNA. The results of the CNA-FWRD registry, as well as other ongoing randomized studies (Efficacy of a Right-sided Ablation of the Anterior Ganglionated Plexus for Neurally Mediated Syncope (CardNMH3) - NCT04755101 - and Cardiac Ganglionated Plexus Ablation Before Permanent Pacemaker Implantation in Patients with Sick Sinus Syndrome (GAPS) - NCT04149886), are awaited to evaluate the long-term safety and effectiveness of CNA.

## 9. Conclusions

CNA is an emerging therapeutic approach for unpredictable and recurrent VVS with cardioinhibitory responses and for functional bradyarrhythmias. Multiple observational studies support lasting benefits in preventing recurrent symptoms and bradycardia. Ongoing studies will shed more light on the safety, efficacy, and long-term benefits of CNA.
